# Mutations and CpG islands among hepatitis B virus genotypes in Europe

**DOI:** 10.1186/s12859-015-0481-8

**Published:** 2015-02-05

**Authors:** Chengyao Zhong, Zhiwei Hou, Jihua Huang, Qingdong Xie, Ying Zhong

**Affiliations:** Faculty of Science and Engineering, University Paul Sabatier, Toulouse, 31062 France; Key Laboratory of Birth Defects and Reproductive Health of National Health and Family Planning Commission, Chongqing Research Institute for Population and Family Planning Science and Technology, Chongqing, 400020 China; Jinxin Research Institute for Reproductive Medicine and Genetics, Chengdu Jinjiang Hospital for Maternal and Child Health Care, Chengdu, 610066 China; Research Center for Reproductive Medicine, Shantou University Medical College, Shantou, 515041 China

**Keywords:** HBV, Genotypes, Mutation, CpG islands, Europe

## Abstract

**Background:**

Hepatitis B virus (HBV) genotypes have a distinct geographical distribution and influence disease progression and treatment outcomes. The purpose of this study was to investigate the distribution of HBV genotypes in Europe, the impact of mutation of different genotypes on HBV gene abnormalities, the features of CpG islands in each genotype and their potential role in epigenetic regulation.

**Results:**

Of 383 HBV isolates from European patients, HBV genotypes A-G were identified, with the most frequent being genotype D (51.96%) in 12 countries, followed by A (39.16%) in 7 countries, and then E (3.66%), G (2.87%), B (1.57%), F (0.52%) and C (0.26%). A higher rate of mutant isolates were identified in those with genotype D (46.7%) followed by G (45.5%), and mutations were associated with structural and functional abnormalities of HBV genes. Conventional CpG island I was observed in genotypes A, B, C, D and E. Conventional islands II and III were detected in all A-G genotypes. A novel CpG island IV was found in genotypes A, D and E, and island V was only observed in genotype F. The A-G genotypes lacked the novel CpG island VI. “Split” CpG island I in genotypes D and E and “split” island II in genotypes A, D, E, F and G were observed. Two mutant isolates from genotype D and one from E were found to lack both CpG islands I and III.

**Conclusions:**

HBV genotypes A-G were identified in European patients. Structural and functional abnormalities of HBV genes were caused by mutations leading to the association of genotypes D and G with increased severity of liver disease. The distribution, length and genetic traits of CpG islands were different between genotypes and their biological and clinical significances warrant further study, which will help us better understand the potential role of CpG islands in epigenetic regulation of the HBV genome.

## Background

Hepatitis B virus (HBV) infection is a serious health problem worldwide. The World Health Organization (WHO) has estimated that around 240 million people globally are chronically infected with HBV, which cause between 500,000 and 700,000 deaths annually [1]. In much of the developing world, (sub-Saharan Africa, most of Asia, and the Pacific), 8% to 10% of people in the general population become chronically infected. Although HBV infection is less common in Western Europe, a recent investigation by the European Centre for Disease Prevention and Control showed an unexpected increase in HBV infection between 1995 and 2005 with an incidence from 0.7% to 5.3% [2,3].

HBV belongs to the genus *Orthohepadnavirus* of the *Hepadnaviridae* family and has a circular genome of approximately 3.2 kb in length. Because of its long history of co-evolution with humans, HBV has evolved as multiple genetic strains that are present at different rates in human populations [4]. Based on a minimum divergence of 8% of the complete genome sequences, HBV is classified as different genotypes consecutively identified as genotypes A–J [4-6]. The genotypes have a distinct geographical distribution. In Europe, genotypes A and D are the main genotypes, and genotype A is more prevalent in northern and central Europe [7], whereas genotype D is mainly found in countries surrounding the Mediterranean Sea and in Eastern Europe [7]. Genotype G was detected in HBV carriers from Germany [8], Netherlands [9] and Georgia [10]. In France, all A-G genotypes were found, the most frequent being genotypes D (27%) and A (24%), followed by E (13%) and C (12%), and B (7%) [11,12]. HBV genotypes are used to trace the evolution and transmission of the virus. Differences between genotypes affect the disease severity, disease course and likelihood of complications, as well as response to treatment and possibly to vaccination [5].

Besides genotypes, specific HBV viral mutations appear to strongly influence the outcome of HBV infection. HBV replicates through reverse transcription of an RNA intermediate. Because the reverse transcriptase activity of the HBV polymerase protein lacks a proofreading function, random mis-incorporation of bases into the replicating DNA strand occurs, leading to a high mutation rate. In addition, mutations occur as a consequence of selection pressure by the host’s immune system and/or by exogenous factors such as active or passive vaccination or drug therapy [6,13].

DNA methylation is being increasingly recognized to play a role in the regulation of viral gene expression. It was demonstrated that HBV DNA can be methylated in human tissue in both a nonintegrated form [14] and following integration into the human genome [15]. DNA methylation typically occurs in a CpG dinucleotide context that is often grouped in clusters called CpG islands. Hypermethylation of CpG islands located in promoter regions are involved in gene silencing at the transcriptional level [16]. The HBV genome contains four promoter elements (sp1, sp2, cp and xp), and two enhancer elements (Enh I and Enh II) that control four defined overlapping open reading frames (pre-S/S, core/e, X, and P genes), which promote transcription and expression of the seven different hepatitis B proteins. Many studies have demonstrated that HBV DNA contains three predicted CpG islands termed conventional CpG islands I, II and III. Recently, Zhang et al. identified three novel CpG islands in 14 of 176 HBV isolates and named them CpG islands IV, V, and VI [6]. Hou et al. also detected CpG islands at the same locations in 30 of 320 HBV isolates from Chinese patients (data not shown).

In the present study, the mutant sequence, as well as the location, distribution, length and genetic traits of CpG islands of different HBV genotypes isolated from the European patients were investigated to help us better understand the impact of mutation on gene expression among genotypes and the potential role of CpG islands in the epigenetic regulation of the HBV genome.

## Methods

Nucleotide sequences of HBV genomes were searched from GenBank using an updated database (last accessed on 31 January 2014) at the National Center for Biotechnology Information (http://www.ncbi.nlm.nih.gov/nucleotide), using “Hepatitis B virus”, “complete genome” and “genotype X” (X represents A, B, C, D, E, F or G) as the terms for search query. Full-length HBV genome sequences of genotypes A through G from European countries were retrieved. The background information consisted of an assessment of genome length, regions, genotype, resource, open reading frame (ORF) location and gene function. HBV genome sequences were manually excluded from the database when they were shorter than 3050 base pairs (bp), or had incomplete background information [17].

Multiple alignments of the 383 HBV sequences, containing complete functional genes or defined gene mutations, were conducted using the CLUSTALX 1.83 program (UCD Conway Institute, Dublin, Ireland). The genotype of each analyzed sequence was compared with the original report to confirm the background genotyping information. If a discrepancy existed between the observed data and the original report, updated information was collected for further analysis.

CpG islands were analyzed using MethPrimer (http://www.urogene.org/methprimer/index1.html) by examining the GC content and the observed-to-expected ratio in a window size. The location and size of CpG islands within each analyzed sequence were identified according to the current knowledge of CpG island distributions in the HBV genomes [18]. Because each genotype contains multiple sequences and the lengths of the sequences are different, we calculated the length of CpG island of each genotype according to their location in the sequence: the most proximal site of the CpG island in the sequence was designated the start and the most distal site was designated the end. To identify each CpG island, the following criteria were defined: a GC content of 0.50 or greater; an observed-to-expected CpG dinucleotide ratio of 0.60 or greater; and both occurring within a sequence window of 100 bps or greater [6,14,19].

## Results

### Geographical distribution of HBV genotypes in Europe

**Figure 1:**
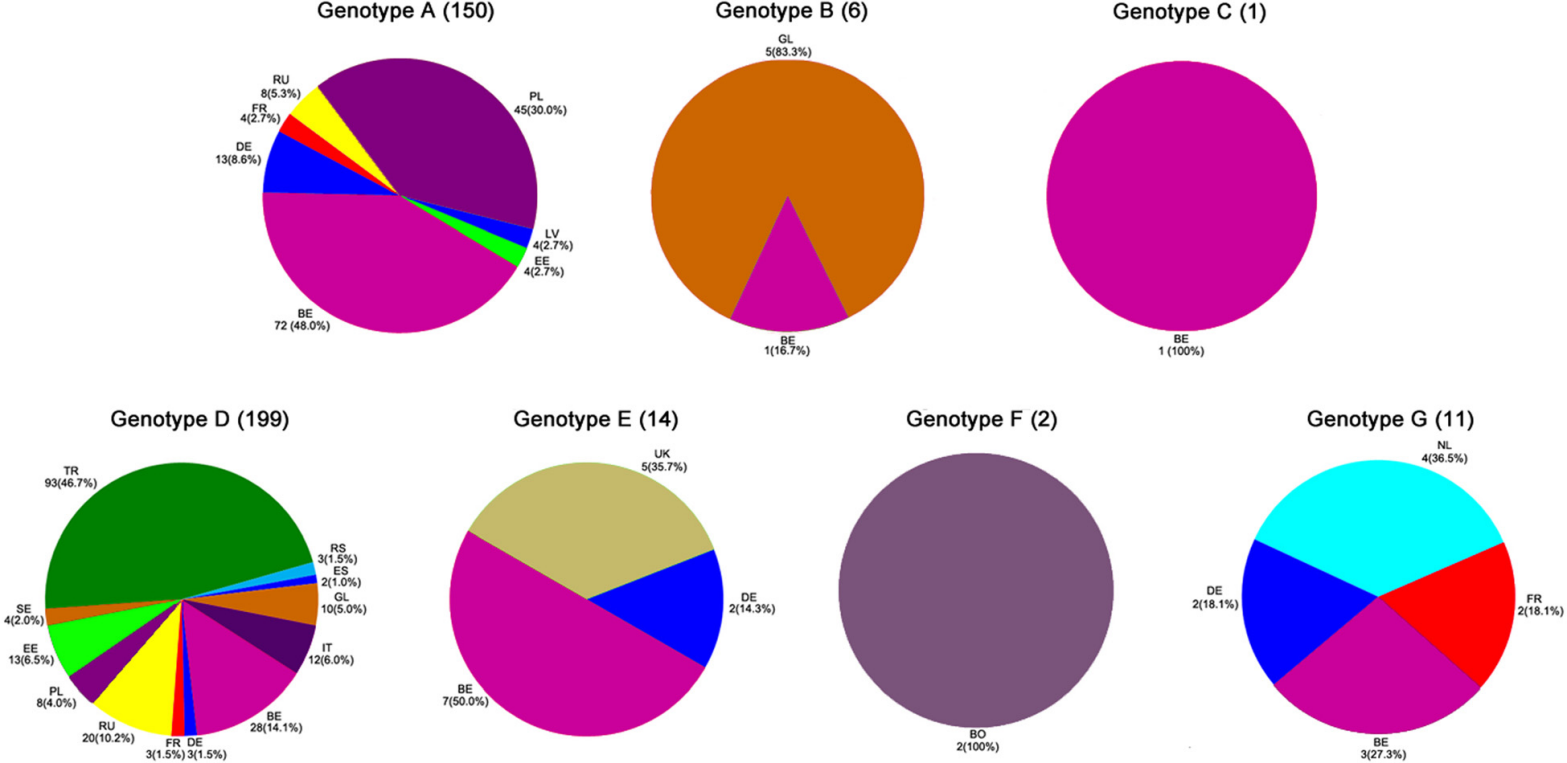
**Geographical distribution of HBV genotypes in some European countries.** BE: Belgium, BO: Bolivia, DE: Germany, EE: Estonia, ES: Spain, FR: France, GL: Greenland, IT: Italy, LV: Latvia, NL: Netherland, PL: Poland, RS: Serbia, RU: Russia, SE: Sweden, TR: Turkey, UK: United Kingdom.

A total of 383 HBV isolates were identified as 7 datasets: genotype A (n = 150, 39.16%), B (n = 6, 1.57%), C (n = 1, 0.26%), D (n = 199, 51.96%), E (n = 14, 3.66%), F (n = 2, 0.52%) and G (n = 11, 2.87%). Genotype A isolates were observed in 7 countries including Belgium (n = 72, 48%), Poland (n = 45, 30%), Germany (n = 13, 8.6%), Russia (n = 8, 5.3%), France (n = 4, 2.7%), Latvia (n = 4, 2.7%) and Estonia (n = 4, 2.7%). Genotype D isolates were from 12 countries including Turkey (n = 93, 46.7%), Belgium (n = 28, 14.1%), Russia (n = 20, 10.2%), Estonia (n = 13, 6.5%), Italy (n = 12, 6.0%), Greenland (n = 10, 5.0%), Poland (n = 8, 4.0%), Sweden (n = 4, 2.0%), Germany (n = 3, 1.5%), France (n = 3, 1.5%), Serbia (n = 3, 1.5%) and Spain (n = 2, 1.0%). Genotypes B, C, E, F and G were from 2, 1, 4, 1 and 4 countries, respectively. Detailed geographical distribution and rates of HBV genotypes in different European countries are shown in Figure 1.

### Mutations among HBV genotypes in Europe

Genotype A consisted of 123 normal and 27 mutant isolates. Genotype B consisted of 6 normal isolates. Genotype C consisted of 1 normal isolate. Genotype D consisted of 106 normal and 93 mutant isolates. Genotype E consisted of 12 normal and 2 mutant isolates. Genotype F consisted of 2 normal isolates. Genotype G consisted of 6 normal and 5 mutant isolates. Genotype A isolates were mainly from Belgium (68 normal and 4 mutant isolates) and Poland (26 normal and 19 mutant isolates), while genotype D isolates were mainly from Turkey (32 normal and 61 mutant isolates) and Belgium (10 normal and 18 mutant isolates). Detailed distribution of the normal and mutant isolates in different European countries is shown in Table 1.

**Table 1:** **The normal and mutant isolates in the different genotypes in some countries in Europe**

**Countries**	**Genotype A**		**Genotype B**		**Genotype C**		**Genotype D**		**Genotype E**		**Genotype F**		**Genotype G**	
	**Nor**	**Mut**	**Nor**	**Mut**	**Nor**	**Mut**	**Nor**	**Mut**	**Nor**	**Mut**	**Nor**	**Mut**	**Nor**	**Mut**
Belgium	68	4	1	-	1	-	10	18	5	2	-	-	-	3
Bolivia	-	-	-	-	-	-	-	-	-	-	2	-		
Estonia	4	-	-	-	-	-	12	1	-	-	-	-	-	-
France	4	-	-	-	-	-	2	1	-	-	-	-	1	1
Germany	10	3	-	-	-	-	1	2	2	-	-	-	2	-
Greenland	-	-	5	-	-	-	10	-	-	-	-	-	-	-
Italy	-	-	-	-	-	-	12	-	-	-	-	-	-	-
Latvia	3	1	-	-	-	-	-	-	-	-	-	-	-	-
Netherland	-	-	-	-	-	-	-	-	-	-	-	-	3	1
Poland	26	19	-	-	-	-	2	6	-	-	-	-	-	-
Russia	8	-	-	-	-	-	18	2	-	-	-	-	-	-
Serbia	-	-	-	-	-	-	2	1	-	-	-	-	-	-
Spain	-	-	-	-	-	-	2	-	-	-	-	-	-	-
Sweden	-	-	-	-	-	-	3	1	-	-	-	-	-	-
Turkey	-	-	-	-	-	-	32	61	-	-	-	-	-	-
United kingdom	-	-	-	-	-	-	-	-	5	-	-	-	-	-
Sum	123	27	6	0	1	0	106	93	12	2	2	0	6	5

Nor: normal sequence; Mut: mutant sequence.

Some mutant isolates in genotypes A, D, E and G were associated with structural and functional abnormalities of HBV genes, including truncated proteins, nonfunctional proteins, proteins containing amino acid (aa) internal deletions, genes containing a point mutation, and pseudogenes. The number and percentage of mutant isolates with structural or functional abnormalities in each genotype are shown in Table 2.

**Table 2:** **The number and percentage of the mutant isolates with the structural or functional abnormalities in the genotypes**

**Genotypes**	**Mutant isolates with abnormalities**		**Types of abnormalities**
	**No.**	%*	
A	26	96%	(1) The truncated proteins including pre-C/C, pre-S1/pre-S2/S and X proteins; (2) the nonfunctional proteins including pre-C/C, pre-S2/S and X proteins; (3) the proteins containing aa internal deletion including pre-C/C and pre-S1/pre-S2/S proteins; (4) X gene containing a point mutation which resulted in a premature stop codon.
		26/27	
D	27	29%	(1) The truncated proteins including pre-C/C, pre-S1/pre-S2/S and X proteins; (2) the nonfunctional proteins including pre-C/C, pre-S1/pre-S2/S, X proteins and polymerase; (3) the proteins containing aa internal deletion including pre-C/C and pre-S1/pre-S2/S proteins; (4) pre-C/C and X genes containing a point mutation which resulted in a premature stop codon; (5) some pre-C/C genes were pseudogenes.
		27/93	
E	2	100%	(1) The truncated pre-C/C protein; (2) the nonfunctional proteins including pre-C/C, polymerase and large S proteins.
		2/2	
G	4	80%	(1) The truncated X protein; (2) the nonfunctional pre-C/C protein; (3) pre-C/C containing a premature stop codon.
		4/5	

*The percentage is equal to number of the mutant isolates with the structural or functional abnormalities/number of total mutant isolates × 100%.

### Location and length differences of CpG islands among HBV genotypes A-G

**Figure 2:**
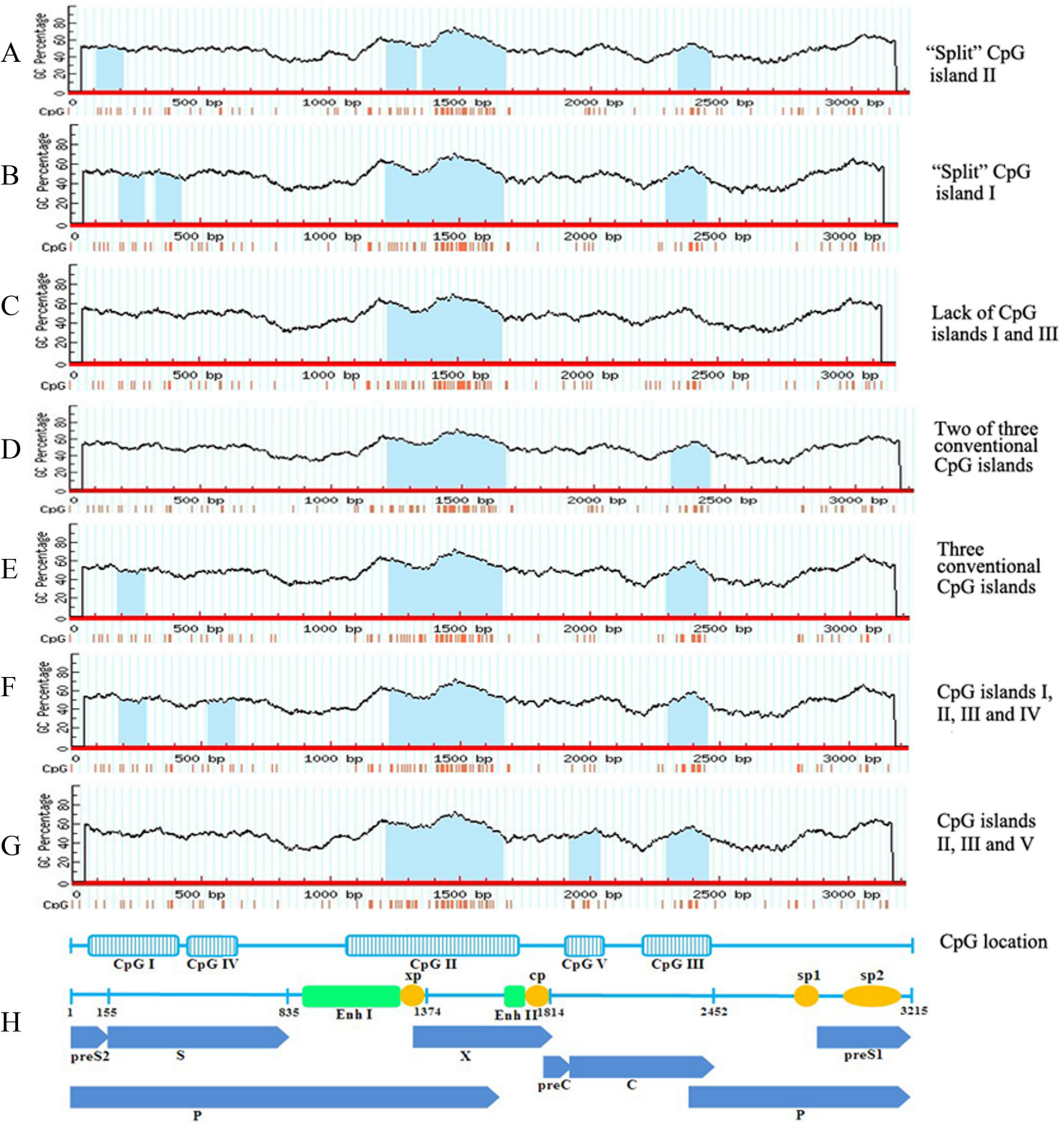
**The CpG island distribution within representative HBV sequences of HBV genotypes A-G.** The open reading frames of the pre-core/core, polymerase, surface antigen, and X genes are indicated as blue arrows. The four promoters, xp, cp, sp1 and sp2, are indicated as solid yellow circles, and the main regulatory elements, enhancers I and II (Enh I and Enh II), are indicated as green boxes. The light blue areas represent the predicted methylation region of CpG islands within the HBV genome. The light blue boxes containing vertical lines represent CpG islands I to V within the HBV genome.

In the present study, CpG island I in A, B, C, D and E genotypes were located at nucleotides 76–291, 108–287, 186–286, 77–432 and 72–286, respectively, which spanned the start site of the S gene. CpG island II in A, B, C, D, E, F and G genotypes were located at nucleotides 1113–1674, 1139–1673, 1219–1663, 1100–1674, 1212–1674, 1216–1673 and 1160–1628, respectively, which covered the partial Enh I and X promoter, located immediately upstream of the Enh II/core gene promoters. CpG island III in A, B, C, D, E, F and G genotypes were located at nucleotides 2185–2466, 2298–2462, 2280–2442, 2188–2465, 2172–2458, 2298–2458 and 2304–2497, respectively, which covered the partial C gene and encompassed the start site of the P gene. CpG island IV in A, D and E genotypes were located at nucleotides 529–632, 443–589, 471–573, respectively, which were located between CpG islands I and II, in a region overlapping the S and P genes [6,20]. Island V was only observed in genotype F, located at nucleotides 1921–2038 and between CpG islands II and III, in a region overlapping the C genes. CpG islands VI was not observed in genotypes A-G (Table 3; Figure 2F-H).

**Table 3:** **The location and length of CpG islands in HBV Genotypes A–G**

**Genotype**	**CpG island I**		**CpG island II**		**CpG island III**		**CpG island IV**		**CpG island V**	
	**Location (bp)**	**Length (bp)**	**Location (bp)**	**Length (bp)**	**Location (bp)**	**Length (bp)**	**Location (bp)**	**Length (bp)**	**Location (bp)**	**Length (bp)**
A	76-291	216	1113-1674	562	2185-2466	282	529-632	104	-	-
B	108-287	180	1139-1673	535	2298-2462	165	-	-	-	-
C	186-286	101	1219-1663	445	2280-2442	163	-	-	-	-
D	77-432	356	1100-1674	575	2188-2465	278	443-589	147	-	-
E	72-286	215	1212-1674	463	2172-2458	287	471-573	103	-	-
F	-	-	1216-1673	458	2298-2458	161	-	-	1921-2038	118
G	-	-	1160-1628	469	2304-2497	194	-	-	-	-

Genotype VI was not observed in all listed genotypes.

### Distribution and genetic trait of CpG islands in HBV genotypes A-G

In a total of 383 isolates, three lacked both CpG island I and III, which were excluded from the distribution analysis (Table 4, Figure 2C). Of the remaining 380 isolates, two normal isolates of genotype F contained both two-conventional islands and novel island V, which were used twice for calculating the rates (Table 4; Figure 2G). The majority of isolates contained the three-conventional CpG islands (I, II and III) (69.21%, 263/380) (Figure 2E), followed by isolates containing the two-conventional CpG islands (II and III) (28.42%, 108/380) (Figure 2D), and then by isolates containing the novel islands (2.89%, 11/380). In genotype B, no mutant isolates and novel CpG islands were observed, while genotype C had only one normal sequence, which contained three-conventional islands. No isolates of genotype G contained the three-conventional and novel islands. The rates of isolates containing different types of islands in the total, normal and mutant groups with genotypes A-G are shown in Table 4.

**Table 4:** **Distribution of CpG islands in HBV genotypes A-G**

**Genotype**	**Three-conventional CpG islands**		**Two-conventional CpG islands**		**Novel CpG islands**	
	**number**	**%**	**number**	**%**	**number**	**%**
A total	115	76.66%	31	20.67%	4	2.67%
(n = 150)						
A normal	100	81.30%	21	17.07%	2	1.63%
(n = 123)						
A mutant	15	55.56%	10	37.04%	2	7.40%
(n = 27)						
B total	4	66.7%	2	33.3%	0	0
(n = 6)						
B normal	4	66.7%	2	33.3%	0	0
(n = 6)						
B mutant	0	0	0	0	0	0
(n = 0)						
C total	1	100%	0	0	0	0
(n = 1)						
C normal	1	100%	0	0	0	0
(n = 1)						
C mutant	0	0	0	0	0	0
(n = 0)						
D total	135	68.53%	59	29.95%	3	1.52%
(n = 197)*						
D normal	71	66.98%	34	32.08%	1	0.94%
(n = 106)						
D mutant	64	70.33%	25	27.47%	2	2.20%
(n = 91)*						
E total	8	61.54%	3	23.08%	2	15.38%
(n = 13)*						
E normal	8	66.66%	2	16.67%	2	16.67%
(n = 12)						
E mutant	0	0	1	100%	0	0
(n = 1)*						
F total	0	0	2	100%	2	100%
(n = 2)						
F normal	0	0	2	100%	2	100%
(n = 2)						
F mutant	0	0	0	0	0	0
(n = 0)						
G total	0	0	11	100%	0	0
(n = 11)						
G normal	0	0	6	100%	0	0
(n = 6)						
G mutant	0	0	5	100%	0	0
(n = 5)						
Sum	263	69.21%	108	28.42%	11**	2.89%

*In a total 383 isolates, three (two mutant isolates from genotype D and one from E) lacked both CpG island I and III, which were excluded from the distribution analysis.

**Of the remaining 380 isolates, two normal isolates of genotype F contained both two-conventional islands and novel island V, which were used twice for calculating the rates.

In 77 of 383 (20.13%) isolates, CpG islands II was interrupted by an unmethylated region (6–30 bps) to form two sections, we named "split" CpG island II (Figure 2A). In genotype A, D, E, F and G, the rates of "split" island II were 2% (3/150), 30.2% (60/199), 28.6% (4/14), 50% (1/2) and 90.9% (10/11), respectively (Table 5). This “split” phenomenon was also detected in CpG island I from 8 isolates (2.1%) (Figure 2B). In genotype D and E, the rates of "split" island I were 3.5% (7/199) and 7.1% (1/14), respectively (Table 5). In addition, two mutant isolates of genotype D (GQ477456 and AB674414) and one mutant isolate of genotype E (GU563552) lacked both CpG islands I and III (Table 5). A nonfunctional pre-C/C protein was observed in GQ477456, and nonfunctional pre-C/C, polymerase and large/middle S proteins were detected in GU563552. In AB674414, the “misc_feature”, a region of biological significance, was detected.

**Table 5:** **The trait analysis of CpG islands in HBV genotypes A-G**

**Genotype**	**“Split” rates**		**Lack rates**
	**CpG island I**	**CpG island II**	**CpG islands I & III**
A	-	2.0% (3/150)	-
B	-	-	-
C	-	-	-
D	3.5% (7/199)	30.2% (60/199)	1.0% (2/199)
E	7.1% (1/14)	28.6% (4/14)	7.1% (1/14)
F	-	50.0% (1/2)	-
G	-	90.9% (10/11)	-

### Comparison of CpG island length between normal and mutant isolates

In genotype A, the length differences (LDs) of islands I, II, III and IV between the normal and mutant isolates were 8, 128, 14 and 1 bp, and the percent differences (PDs) of normal CpG length were 3.85%, 22.7%, 4.96% and 0.96%, respectively. In genotype D, LDs of islands I, II, III and IV were 254, 128, 99 and 45 bp, and the PDs were 249%, 22.3%, 55.31% and 44.12%, respectively. In genotype E, the LDs of islands II and III were 24, and 175 bp, and the PDs were 5.2% and 61.4%, respectively. In genotype G, the LDs of islands II and III were 3 and 46 bp, and the PDs were 0.64% and 23.71%, respectively (Table 6). Functional or structural abnormalities of HBV genes were observed only in mutant isolates with either LD of CpG islands or those without LD (Table 7).

**Table 6:** **The length differences of CpG islands between normal and mutant isolates in HBV Genotypes A-G**

**Genotype**	**CpG island I**			**CpG island II**			**CpG island III**			**CpG island IV**			**CpG island V**		
	**Location (bp)**	**Length (bp)**	**LD and PD**	**Location (bp)**	**Length (bp)**	**LD and PD**	**Location (bp)**	**Length (bp)**	**LD and PD**	**Location (bp)**	**Length (bp)**	**LD and PD**	**Location (bp)**	**Length (bp)**	**LD and PD**
A (n = 123) Normal	78-285	208	8 (3.85%)	1113-1676	564	128 (22.7%)	2185-2466	282	14 (4.96%)	529-632	104	1 (0.96%)	-	-	-
A (n = 27) Mutant	76-291	216		1228-1663	436		2196-2463	268		530-632	103		-	-	
B (n = 6) Normal	108-287	180	-	1139-1683	545	-	2298-2462	165	-	-	-	-	-	-	-
B (n = 0) Mutant	-	-		-	-		-	-		-	-		-	-	
C (n = 1) Normal	186-286	101	-	1219-1663	445	-	2280-2442	263	-	-	-	-	-	-	-
C (n = 0) Mutant	-	-		-	-		-	-		-	-		-	-	
D (n = 106) Normal	185-286	102	254 (249%)	1100-1674	575	128 (22.3%)	2265-2443	179	99 (55.31%)	454-555	102	45 (44.12%)	-	-	-
D (n = 93) Mutant	77-432	356		1191-1637	447		2188-2465	278		443-589	147		-	-	
E (n = 12) Normal	72-286	215	-	1212-1674	463	24 (5.2%)	2172-2456	285	175 (61.4%)	471-583	113	-	-	-	-
E (n = 2) Mutant	-	-		1230-1668	439		2334-2443	110		-	-		-	-	
F (n = 2) Normal	-	-	-	1216-1673	458	-	2298-2458	161	-	-	-	-	1921-2038	118	-
F (n = 0) Mutant	-	-		-	-		-	-		-	-		-	-	
G (n = 6) Normal	-	-	-	1160-1628	469	3 (0.64%)	2304-2497	194	46 (23.71%)	-	-	-	-	-	-
G (n = 5) Mutant	-	-		1163-1628	466		2350-2497	148		-	-		-	-	

LD: the length difference of CpG islands between the normal and mutant isolates; PD: the percent difference in normal CpG length (LD/CpG length of normal isolate x 100%).

**Table 7:** **The relationship between the abnormalities and length differences of CpG islands and mutations in HBV Genotypes A-G**

**Genotype**	**Island I**		**Island II**		**Island III**		**Island IV**		**Island V**	
	**LD-Y**	**LD-N**	**LD-Y**	**LD-N**	**LD-Y**	**LD-N**	**LD-Y**	**LD-N**	**LD-Y**	**LD-N**
A	+	+	-	+	-	+	-	+	-	-
D	+	+	-	+	+	+	+	-	-	-
E	-	-	-	+	-	+	-	+	-	-
G	-	-	-	+	-	+	-	-	-	-

All functional or structural abnormalities of HBV genes are observed in the mutant isolates not in the normal isolates.

LD-Y: lengths of CpG islands between normal and mutant isolates are different. LD-N: lengths of CpG islands between normal and mutant isolates are not different. +: The functional or structural abnormalities of HBV genes are observed; −: The functional or structural abnormalities of HBV genes are not observed.

## Discussion

### Geographical distribution of HBV genotypes in Europe

Of the 383 HBV isolates obtained from European patients, the most frequent was genotype D, followed by genotype A, in accordance with the previous reports on the geographical distribution of HBV genotypes in Europe, especially the western part of Europe [7,10-12,21-23]. Genotype A was observed in 7 countries and genotype D in 12 countries. All other minor occurring genotypes including B, C, E, F and G, were found in 7 countries (Figure 1). Of these countries, Belgium had the greatest genotypic variation (A, B, C, D, E and G). The highest rates of HBV isolates in all genotypes (29.24%, 112/383), especially genotype A (48%, 72/150) were also found in Belgium. This might be explained by recent immigration from Far East Asia and Africa to Europe [24] and indicates Belgium, as a Western European country, has had an increased rate of HBV infections during the last decade [2]. For genotype D, the highest rate of HBV isolates was found in Turkey, which might be because Turkey has served as a bridge for many migration events during history [23].

### Mutations among genotypes in Europe

In the present study, the highest rates of mutant isolates occurred in genotypes D (46.7%) and G (45.5%). These mutations were associated with structural or functional abnormalities of the HBV genes. In genotype D, most significant functional abnormalities occurred in the pre-C/C region that include the truncated pre-C/C protein, the nonfunctional pre-C/C protein, or pre-C/C proteins containing an aa internal deletion. These abnormal changes might result from a point mutation occurring in the pre-C/C region or from pre-C/C genes becoming pseudogenes. It was shown that pre-C/C genes in some isolates of genotype D contained a point mutation at position 1896 (G to A) that resulted in a premature stop codon (TGG to TAG) at the end of the precore region, preventing Hepatitis B e antigen (HBeAg) synthesis [25]. Lack of circulating HBeAg, an immune tolerogen, might contribute to a more aggressive disease and cause liver damage with progression to cirrhosis and cancer or fulminant hepatitis [4,17,25]. This might explain why genotype D is associated with more severe liver diseases [4]. Genotype G infection was often detected in the context of co-infection with human immunodeficiency virus (HIV) or recombination with genotype A [26]. It is characterized by stop codons at codon 2 and 28 of the pre-C region and insertion of 36 nucleotides at the 5' end of the C gene [8] and by mutations preventing expression of HBeAg [27]. In the present study, 54.5% (6/11) of genotype G isolates were from patients co-infected with HIV. The mutations occurred in the pre-C/C and X gene regions and led to the nonfunctional pre-C/C protein and the truncated X proteins, respectively. The frequent finding of genotype G in co-infected patients and its association with more advanced fibrosis suggests this genotype leads to rapid liver disease progression [28].

### Comparison of CpG island length between normal and mutant isolates

Some studies have reported that the lengths of CpG islands in mammalian genomes exhibit substantial variation. Because promoters with long CpG islands have a larger number of RNA polymerase II binding sites compared to promoters with short CpG islands, it was proposed that variations in the length of CpG island promoters is tightly linked to patterns of downstream gene expression [29]. In the present study, the LDs of CpG islands between the normal and mutant isolates were observed in genotypes A, D, E and G. Although the functional abnormalities of HBV genes were detected in some of the mutant isolates with LDs of islands, such abnormalities were also found in some mutant isolates without island LDs. Because all abnormalities were observed in the mutant isolates, such abnormalities should be caused by a mutation in isolates but not by the LD of the island. However, the biological and clinical significance of LD of islands warrants further study as islands located in the regulatory region are critical for transcription.

### Novel CpG islands in HBV genotypes A-G

The three novel CpG islands in the HBV genome were first described by Zhang et al., and were thought to be potential targets for DNA methylation [6]. We also found novel islands at the same locations in the HBV genome. In our study, novel island IV was present in genotypes A, D and E, island V was present in genotype F; and island VI was not present in A-G genotypes. In the report by Zhang et al., island IV was detected in genotypes B, C and D, island V in genotypes B, F and H, and island VI in genotype C. The reason for differences between both studies might be that data was obtained from different sources. Our data were from 383 isolates of European patients, and Zhang's data was from 176 representative isolates of patients residing in 48 different countries worldwide. In our study, island IV was located between CpG islands I and II and upstream of Enh I, a region overlapping the S and P gene, island V was located between islands II and III, close to Enh II and the core promoters, a region overlapping the C genes. Based on their position, their methylation status might be associated with the transcription of HBV genes. To date, there is no experimental data on the relationship between the methylation status of novel islands and expression of HBV genes, which requires further study.

### Distribution and genetic trait of CpG islands in HBV genotypes A-G

The various distributions of CpG islands in different genotypes were observed in this study. Island I was observed in genotypes A, B, C, D and E, and islands II and III were detected in all A-G genotypes. These results were similar to a previous study [6]. The reason for some isolates only containing islands II and III might be the density of CpG dinucleotides within the first CpG-rich region in those sequences being low, leading to a loss of island I [6]. Because HBV DNA is methylated in human tissues either as a nonintegrated or integrated form [14,15] and DNA methylation is CpG site-specific [30], the density and location of HBV CpG islands could have a direct impact on HBV gene expression in human tissue through their methylation.

We found “split” CpG islands in 77 of 383 (20.13%) isolates of genotypes A, D, E, F and G. This “split” phenomenon was also detected in genotypes B and C isolated from Chinese patients in our previous research (data not shown). The occurrence mechanisms and the clinical significance of “split” CpG islands I or II remain unclear. In this study we found that two mutant isolates of genotype D (GQ477456 and AB674414) and one mutant isolate from genotype E (GU563552) lacked both islands I and III (Table 5). Island II is located in a regulatory region critical for the transcription of covalently closed circular DNA (cccDNA), island I spans the start site of the S gene, and island III covers the partial C gene and encompasses the start site of the P gene, therefore by investigating their methylation and mutation status, further insights into the mechanism of regulatory complexity of gene expression and clinicopathology might be obtained.

## Conclusions

HBV genotypes A-G were identified in 383 isolates from European patients and the most frequent were genotypes D and A, followed by E, G, B, F and C. Higher rates of mutant isolates were observed in genotypes D and G, and isolates lacking both CpG islands I and III were observed in genotypes D and E. Mutations in the genotypes might affect the regulation of HBV gene expression, leading to different clinical outcomes. The findings in this study will aid our understanding of the impact of mutation on HBV gene expression among genotypes and the potential role of CpG islands in the epigenetic regulation of the HBV genome. A limitation of the current work was the small number of isolates in some genotypes, especially genotypes B, C and F, which should be cumulatively increased to obtain more complete information.
